# Personal

**Published:** 1894-10

**Authors:** 


### Personal.

DR. THOMAS B. CARPENTER, of 660 Main street, Buffalo, announces that he has fitted up a laboratory in connection with his office, where he is prepared to make analyses of urine, as well as microscopical examinations of sputum and the various pathological discharges.

Dr. J. F. Krug, of Buffalo, and a member of the board of censors of the Medical Society of the County of Erie, states that he has been made the victim of the unfortunate advertising craze in a weekly newspaper printed in the Polish language and published in this city.

It appears that Mr. A. Karwowski, publisher of the *Reforma*, inserted the advertisement referred to without Dr. Krug's knowledge or consent. Mr. Karwowski has made acknowledgment of this fact in the daily newspapers, which clears Dr. Krug of any suspicion in the premises.

---

Dr. Julius Pohlman, of Buffalo, who assumed the practice of Dr. George M. Gould, of Philadelphia, during the absence of the latter in Europe, has returned home and taken up his own practice of ophthalmology. His offices are, as before, at 367 Franklin street.

---

Dr. Charles A. Ring, who removed to Johnson's Creek, Niagara county, N. Y., some time ago, has returned to Buffalo and established his offices at 128 Fargo avenue.

---

Dr. A. A. Hubbell, of Buffalo, who with his family has been some weeks in Europe, has returned and resumed his professional work.

---

Dr. William C. Krauss, of 382 Virginia street, Buffalo, returned from his European trip September 18, 1894.

